# Crystal structure of 1,1′-(pyridine-2,6-di­yl)bis­[*N*-(pyridin-2-ylmeth­yl)methanaminium] dichloride dihydrate

**DOI:** 10.1107/S205698902101183X

**Published:** 2021-11-16

**Authors:** Layachi Merabet, Marine Tassé, Sonia Mallet-Ladeira, Lakhemici Kaboub, Isabelle Malfant

**Affiliations:** a Laboratoire de Chimie de Coordination, UPR-CNRS 8241, 205 route de Narbonne, 31077 Toulouse cedex, France; b Laboratory of Chemistry, Molecular Engineering and Nanostructures, Department of Chemistry, Faculty of Sciences, University of Ferhat Abbas-Sétif -1, 19000 Sétif, Algeria; c Institut de Chimie de Toulouse UAR-CNRS 2599, 118 route de Narbonne, 31062 Toulouse Cedex 09, France

**Keywords:** crystal structure, hydrogen bonding, π–π inter­actions

## Abstract

In the title compound, C_19_H_23_N_5_^2+^·2Cl^−^·2H_2_O, the two pyridine side arms are not coplanar, with the terminal pyridine rings subtending a dihedral angle of 26.45 (6)°. In the crystal, hydrogen bonds, inter­molecular C—H⋯Cl contacts and a weak C—H⋯O inter­action connect the mol­ecule with neighbouring chloride counter-anions and lattice water mol­ecules. The crystal packing also features by π–π inter­actions with centroid-centroid distances of 3.4864 (12) and 3.5129 (13) Å.

## Abstract 2

In the title compound,the two pyridine side arms are not coplanar, with the terminal pyridine rings subtending a dihedral angle of 26.45 (6)°. In the crystal, hydrogen bonds, inter­molecular C—H⋯Cl contacts and a weak C—H⋯O inter­action connect the mol­ecule with neighbouring chloride counter-anions and lattice water mol­ecules. The crystal packing also features by π–π inter­actions.

## Chemical context

In recent years, ruthenium nitrosyl complexes have attracted considerable attention, essentially because of their inter­esting photoreactivity properties such as photochromism (Schaniel *et al.*, 2007 ▸) and nitric oxide photorelease (Rose & Mascharak, 2008*a* ▸). Ruthenium nitrosyl complexes could have desirable photoreactivity properties relying on the nature of the ligands. The utilization of polydentate ligands in coordination chemistry gives a few benefits over monodentate ligands, in particular because of the chelate effect (Martell, 1967 ▸). Multidentate pyridyl­amine derivative ligands can better control the stability (Afshar *et al.*, 2004 ▸; Eroy-Reveles *et al.*, 2007 ▸), solubility (Harrop *et al.*, 2005 ▸) and structural characteristics of the resulting complex. More particularly, ruthenium complexes derived from penta­dentate ligands are generally stable in physiological media (Halpenny *et al.*, 2007 ▸; Rose & Mascharak, 2008*b* ▸). This stability is necessary for (i) maintaining pharmacological activity, (ii) reducing the toxicity of free metal ions, and (iii) avoiding non-specific binding of partially connected metal ions with other biomolecules (Fry & Mascharak, 2011 ▸; Hoffman-Luca *et al.*, 2009 ▸; Patra & Mascharak, 2003 ▸; Heilman *et al.*, 2012 ▸). In the search for new systems, we report here the synthesis and crystal structure of 1,1′-(pyridine-2,6-di­yl)bis­[*N*-(pyridin-2-ylmeth­yl)methan­am­in­ium] dichloride dihydrate, which contains multiple coord­ination sites, and is thus an excellent candidate for forming stable ruthenium nitrosyl complexes.

## Structural commentary

**Figure 1:**
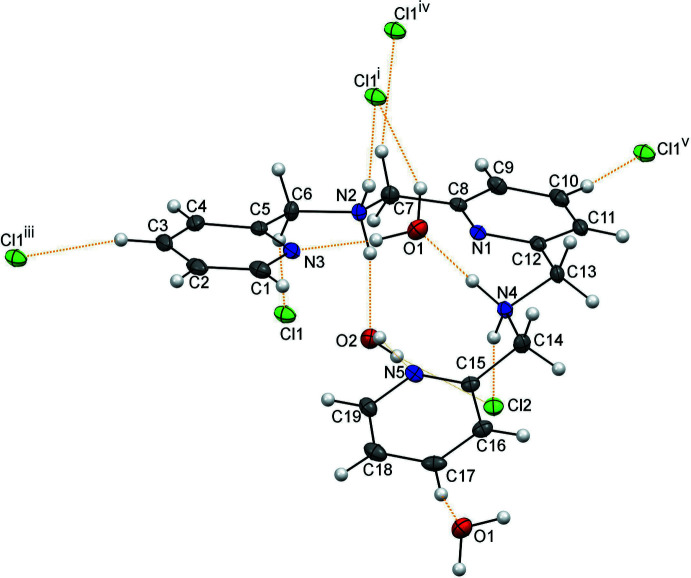
Mol­ecular structure showing the atom-labelling scheme. Displacement ellipsoids are drawn at the 50% probability level. H atoms are drawn as small spheres of an arbitrary radius. The orange dashed lines represent hydrogen bonds, C—H⋯Cl inter­actions and the weak C—H⋯O inter­action. [Symmetry codes: (i) *x* − 1, *y*, *z*; (iii) *x* − 1, *y* + 1, *z*; (iv) −*x* + 1, −*y* + 1, −*z* + 2; (v) *x*, *y* − 1, *z*.]

The title compound crystallizes in the triclinic space group *P* 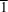 with one cationic mol­ecule, two chloride anions, and two water mol­ecules per asymmetric unit. In the organic mol­ecule, one terminal pyridine ring is almost co-planar with the central pyridine ring, making a dihedral angle of 4.56 (8)°, while the second terminal pyridine ring is out of the plane with a dihedral angle between the two terminal pyridine rings of 26.45 (6)° (Fig. 1 ▸). Bond lengths are within normal ranges and comparable with values found for a similar compound, *N*,*N*′-dialkyl-2,6-pyridine­dimethanaminium (Kobayashi *et al.*, 2006 ▸). 
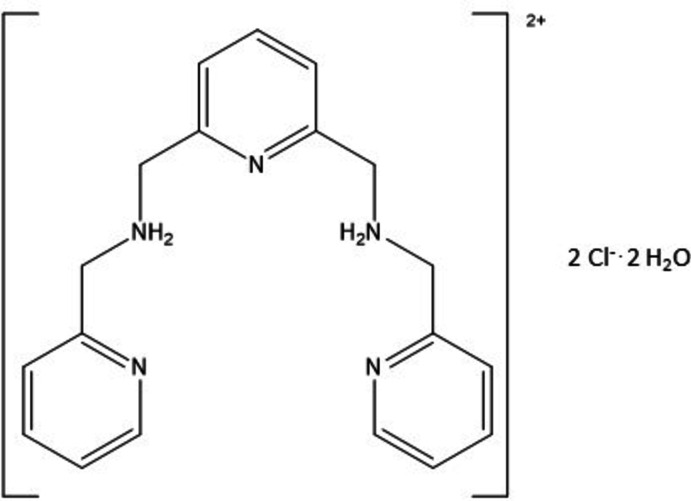


## Supra­molecular features

In the crystal, there are inter­molecular hydrogen bonds (Table 1 ▸) and C—H⋯Cl and C—H⋯O inter­actions between the mol­ecules, the chloride anions and the lattice water mol­ecules. The mol­ecular structure of the compound is illustrated in Fig. 1 ▸ with hydrogen bonding indicated.

**Figure 2:**
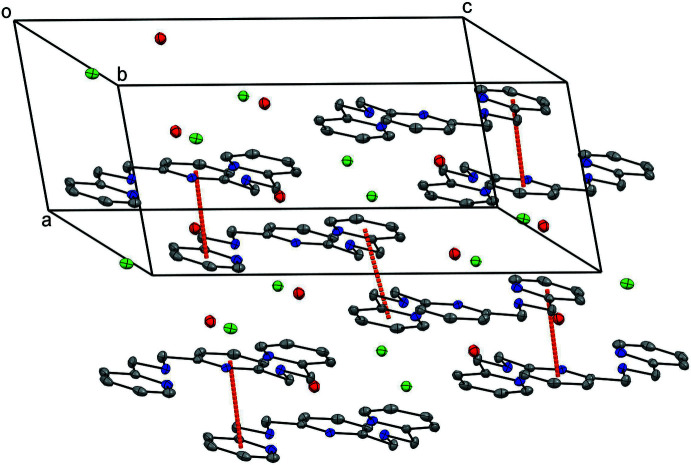
Views of the stacking along the *a* axis. Orange lines indicate π–π inter­actions. Displacement ellipsoids are drawn at the 50% probability level.

The crystal packing shows π–π inter­actions between two parallel neighbouring mol­ecules along the *a*-axis direction with a *Cg*1⋯*Cg*2 (*x*, *y* − 1, *z*) centroid–centroid distance of 3.4864 (12) Å, a perpendicular distance from the centroid *Cg*1 to the plane of the other ring of 3.2472 (8) Å and a slippage between the centroids of 1.100 Å. Similarly, the second π–π stacking inter­action has a *Cg*3⋯*Cg*3(−*x*, −*y* + 2, −*z* + 1) centroid-centroid distance of 3.5129 (13) Å, a perpendicular distance from the centroid *Cg*3 to the plane of the other ring of 3.2177 (8) Å and a slippage between the centroids of 1.410 Å. *Cg*1, *Cg*2 and *Cg*3 are the centroids of N1/C8–C12, N3/C1–C5 and N5/C15–C19 pyridine rings, respectively (Fig. 2 ▸).

## Database survey

A search of the Cambridge Structural Database (CSD, version 5.42, last updated May 2021; Groom *et al.*, 2016 ▸) for similar compounds gave three hits. They include *N,N*’-bis­(2-pyridyl­meth­yl)pyridine-2,6-dicarboxamide (CSD refcode AVURAK; Jain *et al.*, 2004 ▸), *N*,*N*′-bis­[2-(2-pyrid­yl)meth­yl]pyridine-2,6-dicarboxamide hemihydrate (HULKUU; Jian Ying Qi *et al.*, 2002 ▸) and 2,6-bis­[(2-pyridiniometh­yl)ammonio­meth­yl]pyri­dine tetra­chloride monohydrate (IRODAV; Kobayashi *et al.*, 2006 ▸). In those compounds, the two terminal pyridine rings are rotated out of the plane of the central pyridine ring with dihedral angles ranging from 63 to 89°.

## Synthesis and crystallization

1,1′-(Pyridine-2,6-di­yl)bis­[*N*-(pyridin-2-ylmeth­yl)methan­am­inium] dichloride dihydrate compound was obtained following the procedure previously reported in the literature (Gruenwedel, 1968 ▸; Newkome *et al.*, 1984 ▸; Darbre *et al.*, 2002 ▸; Kobayashi *et al.*, 2006 ▸). The procedure used for the synthesis has three steps. Firstly, the synthesis of 2-[(tosyl­amino)­meth­yl]pyridine was carried out by treatment of 2-(amino­meth­yl) pyridine with NaOH and tosyl chloride in a two-phase system (water/diethyl ether) (Newkome *et al.*, 1984 ▸). In the second step, the coupling of 2-[(tosyl­amino)­meth­yl] pyridine with 2,6-bis­(bromo­meth­yl) pyridine, also in an two-phase system (di­chloro­methane/water) and *n*Bu_4_NBr as phase-transfer catalyst gave 2,6-bis­{[(pyrid-2-ylmeth­yl)(tos­yl)amino]­meth­yl}pyridine, which could be isolated after chromatography (Darbre *et al.*, 2002 ▸). Finally, the tosyl­ate group of 2,6-bis­{[(pyrid-2-ylmeth­yl)(tos­yl)amino]­meth­yl}pyridine was removed using concentrated sulfuric acid for deprotection with heating at 393 K for 3 h to give an unstable brownish oil (Newkome *et al.*, 1984 ▸).

Slow diffusion between toluene and a wet di­chloro­methane solution of the brown oil set aside at room temperature gave colourless needles of the title compound suitable for X-ray diffraction within five days.

## Refinement

Crystal data, data collection and structure refinement details are summarized in Table 2 ▸. Hydrogen atoms of the water mol­ecules and those bonded to nitro­gen atoms were located in difference-Fourier maps and refined freely with isotropic displacement parameters. All C-bound H atoms were placed in calculated positions and refined using a riding model, with C—H = 0.95 (aromatic) or 0.99 Å (methyl­ene) and with *U*_iso_(H) = 1.2*U*_eq_(C). For two similar N—H distances, a restraint was applied to make them approximately equal with an effective standard deviation of 0.02 Å.

## Supplementary Material

Crystal structure: contains datablock(s) I. DOI: 10.1107/S205698902101183X/tx2045sup1.cif

Structure factors: contains datablock(s) I. DOI: 10.1107/S205698902101183X/tx2045Isup2.hkl

Supporting information file. DOI: 10.1107/S205698902101183X/tx2045Isup3.cml

CCDC reference: 2120892

Additional supporting information:  crystallographic information; 3D view; checkCIF report

## Figures and Tables

**Table 1:** Hydrogen-bond geometry (Å, °)

*D*—H⋯*A*	*D*—H	H⋯*A*	*D*⋯*A*	*D*—H⋯*A*
O2—H201⋯Cl2	0.85 (3)	2.41 (3)	3.1844 (15)	152 (2)
O2—H202⋯N5	0.91 (3)	2.10 (3)	2.947 (2)	155 (2)
N2—H21⋯O2	0.94 (2)	1.89 (2)	2.804 (2)	165 (2)
N2—H22⋯Cl1^i^	0.86 (2)	2.31 (2)	3.1446 (17)	162.5 (17)
N4—H41⋯Cl2	0.91 (2)	2.25 (2)	3.1319 (17)	164.1 (19)
N4—H42⋯O1^i^	0.97 (2)	1.90 (2)	2.825 (2)	158 (2)
O1—H101⋯Cl1	0.93 (3)	2.43 (3)	3.2611 (15)	149 (2)
O1—H102⋯N3^ii^	1.00 (4)	1.93 (4)	2.920 (2)	172 (3)
C3—H3⋯Cl1^iii^	0.95	2.73	3.5702 (19)	148
C6—H6*A*⋯Cl1	0.99	2.8	3.751 (2)	161
C7—H7*A*⋯Cl1^iv^	0.99	2.78	3.7351 (18)	162
C10—H10⋯Cl1^v^	0.95	2.71	3.6469 (18)	168
C17—H17⋯O1^vi^	0.95	2.57	3.437 (2)	151

Symmetry codes: (i) x-1, y, z; (ii) x+1, y, z; (iii) x-1, y+1, z; (iv) -x+1, -y+1, -z+2; (v) x, y-1, z; (vi) -x+1, -y+2, -z+1.

**Table 2:** Experimental details

Crystal data	
Chemical formula	C_19_H_23_N_5_^2+^·2Cl^−^·2H_2_O
*M*_r_	428.36
Crystal system, space group	Triclinic, *P*\overline{1}
Temperature (K)	110
*a*, *b*, *c* (Å)	7.1579 (6), 8.8119 (7), 17.4150 (13)
α, β, γ (°)	80.357 (3), 80.805 (3), 68.919 (3)
*V* (Å^3^)	1004.52 (14)
*Z*	2
Radiation type	Mo *K*α
μ (mm^−1^)	0.35
Crystal size (mm)	0.2 × 0.08 × 0.04

Data collection	
Diffractometer	Bruker Kappa APEXII Quazar
Absorption correction	Multi-scan (*SADABS*; Krause *et al.*, 2015 ▸)
*T*_min_, *T*_max_	0.660, 0.746
No. of measured, independent and observed [*I* > 2σ(*I*)] reflections	29584, 6970, 4632
*R*_int_	0.097
(sin θ/λ)_max_ (Å^−1^)	0.746

Refinement	
*R*[*F* ^2^ > 2σ(*F* ^2^)], *wR*(*F* ^2^), *S*	0.05, 0.123, 1.03
No. of reflections	6970
No. of parameters	283
No. of restraints	1
H-atom treatment	H atoms treated by a mixture of independent and constrained refinement
Δρ_max_, Δρ_min_ (e Å^−3^)	0.45, −0.38

Computer programs: *APEX3* and *SAINT* (Bruker, 2012 ▸), *SHELXT* (Sheldrick 2015*a* ▸), *SHELXL2018/3* (Sheldrick, 2015*b* ▸), *Mercury* (Macrae *et al.*, 2020 ▸) and *publCIF* (Westrip, 2010 ▸).

## supplementary crystallographic information

### Crystal data

**Table:**

C_19_H_23_N_5_^2+^·2Cl^−^·2H_2_O	*Z* = 2
*M**_r_* = 428.36	*F*(000) = 452
Triclinic, *P*1	*D*_x_ = 1.416 Mg m^−^^3^
Hall symbol: -P 1	Mo *K*α radiation, λ = 0.71073 Å
*a* = 7.1579 (6) Å	Cell parameters from 4444 reflections
*b* = 8.8119 (7) Å	θ = 3.2–31.4°
*c* = 17.4150 (13) Å	µ = 0.35 mm^−^^1^
α = 80.357 (3)°	*T* = 110 K
β = 80.805 (3)°	Needle, colourless
γ = 68.919 (3)°	0.2 × 0.08 × 0.04 mm
*V* = 1004.52 (14) Å^3^	

### Data collection

**Table:**

Bruker Kappa APEXII Quazar diffractometer	6970 independent reflections
Radiation source: microfocus sealed tube	4632 reflections with *I* > 2σ(*I*)
Multilayer optics monochromator	*R*_int_ = 0.097
phi and ω scans	θ_max_ = 32.0°, θ_min_ = 1.2°
Absorption correction: multi-scan (SADABS; Krause *et al.*, 2015)	*h* = −10→10
*T*_min_ = 0.660, *T*_max_ = 0.746	*k* = −13→13
29584 measured reflections	*l* = −25→25

### Refinement

**Table:**

Refinement on *F*^2^	Primary atom site location: dual
Least-squares matrix: full	Secondary atom site location: difference Fourier map
*R*[*F*^2^ > 2σ(*F*^2^)] = 0.05	Hydrogen site location: mixed
*wR*(*F*^2^) = 0.123	H atoms treated by a mixture of independent and constrained refinement
*S* = 1.03	*w* = 1/[σ^2^(*F*_o_^2^) + (0.0518*P*)^2^] where *P* = (*F*_o_^2^ + 2*F*_c_^2^)/3
6970 reflections	(Δ/σ)_max_ = 0.002
283 parameters	Δρ_max_ = 0.45 e Å^−^^3^
1 restraint	Δρ_min_ = −0.38 e Å^−^^3^

### Special details

**Table:**

Geometry. All esds (except the esd in the dihedral angle between two l.s. planes) are estimated using the full covariance matrix. The cell esds are taken into account individually in the estimation of esds in distances, angles and torsion angles; correlations between esds in cell parameters are only used when they are defined by crystal symmetry. An approximate (isotropic) treatment of cell esds is used for estimating esds involving l.s. planes.

### Fractional atomic coordinates and isotropic or equivalent isotropic displacement parameters (Å^2^)

**Table:**

	*x*	*y*	*z*	*U*_iso_*/*U*_eq_	
C1	−0.0133 (3)	1.1436 (2)	0.77592 (11)	0.0187 (4)	
H1	−0.057178	1.157686	0.725689	0.022*	
C2	−0.0514 (3)	1.2816 (2)	0.81173 (12)	0.0209 (4)	
H2	−0.118646	1.387618	0.786614	0.025*	
C3	0.0110 (3)	1.2617 (2)	0.88529 (12)	0.0209 (4)	
H3	−0.013857	1.353959	0.91186	0.025*	
C4	0.1098 (3)	1.1057 (2)	0.91921 (11)	0.0177 (4)	
H4	0.15363	1.089007	0.969628	0.021*	
C5	0.1446 (3)	0.9729 (2)	0.87872 (10)	0.0141 (3)	
C6	0.2661 (3)	0.8058 (2)	0.91424 (10)	0.0154 (3)	
H6A	0.405015	0.802589	0.916169	0.018*	
H6B	0.20699	0.78592	0.968744	0.018*	
C7	0.3958 (3)	0.5088 (2)	0.90648 (10)	0.0152 (3)	
H7A	0.32656	0.482715	0.958206	0.018*	
H7B	0.528732	0.510415	0.914788	0.018*	
C8	0.4252 (3)	0.3788 (2)	0.85503 (10)	0.0123 (3)	
C9	0.5158 (3)	0.2141 (2)	0.88081 (10)	0.0159 (4)	
H9	0.555649	0.180246	0.93221	0.019*	
C10	0.5467 (3)	0.1000 (2)	0.82986 (11)	0.0169 (4)	
H10	0.610739	−0.013533	0.84539	0.02*	
C11	0.4830 (3)	0.1539 (2)	0.75619 (10)	0.0153 (3)	
H11	0.502104	0.078354	0.720177	0.018*	
C12	0.3904 (3)	0.3211 (2)	0.73580 (10)	0.0126 (3)	
C13	0.3100 (3)	0.3899 (2)	0.65770 (10)	0.0143 (3)	
H13A	0.397572	0.323319	0.616689	0.017*	
H13B	0.172891	0.385542	0.659994	0.017*	
C14	0.2369 (3)	0.6343 (2)	0.55940 (10)	0.0155 (3)	
H14A	0.096005	0.640498	0.55942	0.019*	
H14B	0.321889	0.562201	0.519947	0.019*	
C15	0.2502 (3)	0.8035 (2)	0.53686 (10)	0.0134 (3)	
C16	0.2354 (3)	0.8748 (2)	0.45938 (10)	0.0161 (4)	
H16	0.225967	0.815293	0.420322	0.019*	
C17	0.2348 (3)	1.0342 (2)	0.44024 (11)	0.0186 (4)	
H17	0.225559	1.085635	0.387808	0.022*	
C18	0.2479 (3)	1.1176 (2)	0.49930 (11)	0.0197 (4)	
H18	0.245914	1.227408	0.488339	0.024*	
C19	0.2640 (3)	1.0358 (2)	0.57442 (11)	0.0169 (4)	
H19	0.273735	1.092671	0.614568	0.02*	
N1	0.3620 (2)	0.43202 (17)	0.78433 (8)	0.0120 (3)	
N2	0.2743 (2)	0.67297 (17)	0.87003 (9)	0.0127 (3)	
N3	0.0822 (2)	0.99031 (18)	0.80798 (9)	0.0160 (3)	
N4	0.3036 (2)	0.56233 (17)	0.63771 (8)	0.0129 (3)	
N5	0.2668 (2)	0.88122 (17)	0.59397 (8)	0.0146 (3)	
Cl1	0.82559 (7)	0.68542 (5)	0.91223 (3)	0.01828 (11)	
Cl2	0.76725 (7)	0.49614 (5)	0.62355 (3)	0.01743 (11)	
O1	0.9786 (2)	0.76904 (17)	0.72866 (8)	0.0210 (3)	
O2	0.4905 (2)	0.76661 (17)	0.73277 (8)	0.0205 (3)	
H201	0.592 (4)	0.689 (3)	0.7158 (14)	0.038*	
H202	0.389 (4)	0.807 (3)	0.7017 (16)	0.05*	
H21	0.345 (3)	0.689 (2)	0.8209 (11)	0.018 (5)*	
H22	0.152 (3)	0.676 (2)	0.8706 (11)	0.013 (5)*	
H41	0.431 (4)	0.562 (3)	0.6375 (13)	0.026 (6)*	
H42	0.216 (4)	0.622 (3)	0.6789 (12)	0.037 (7)*	
H101	0.917 (4)	0.721 (3)	0.7716 (15)	0.040 (7)*	
H102	1.026 (5)	0.841 (4)	0.7531 (19)	0.084 (11)*	

### Atomic displacement parameters (Å^2^)

**Table:**

	*U*^11^	*U*^22^	*U*^33^	*U*^12^	*U*^13^	*U*^23^
C1	0.0158 (9)	0.0187 (9)	0.0217 (9)	−0.0080 (7)	−0.0011 (7)	0.0014 (7)
C2	0.0147 (9)	0.0153 (8)	0.0302 (10)	−0.0057 (7)	0.0053 (8)	−0.0016 (7)
C3	0.0187 (10)	0.0150 (8)	0.0291 (10)	−0.0080 (7)	0.0087 (8)	−0.0084 (7)
C4	0.0159 (9)	0.0194 (9)	0.0187 (9)	−0.0077 (7)	0.0042 (7)	−0.0068 (7)
C5	0.0121 (9)	0.0145 (8)	0.0164 (8)	−0.0062 (7)	0.0024 (7)	−0.0031 (6)
C6	0.0184 (9)	0.0148 (8)	0.0142 (8)	−0.0053 (7)	−0.0028 (7)	−0.0047 (6)
C7	0.0179 (9)	0.0132 (8)	0.0133 (8)	−0.0039 (7)	−0.0046 (7)	0.0012 (6)
C8	0.0109 (8)	0.0136 (7)	0.0136 (8)	−0.0062 (6)	−0.0010 (6)	−0.0002 (6)
C9	0.0164 (9)	0.0144 (8)	0.0173 (8)	−0.0061 (7)	−0.0060 (7)	0.0032 (6)
C10	0.0135 (9)	0.0112 (8)	0.0240 (9)	−0.0035 (7)	−0.0021 (7)	0.0016 (7)
C11	0.0147 (9)	0.0122 (8)	0.0202 (9)	−0.0061 (7)	0.0005 (7)	−0.0038 (6)
C12	0.0099 (8)	0.0121 (7)	0.0160 (8)	−0.0043 (6)	−0.0005 (6)	−0.0015 (6)
C13	0.0180 (9)	0.0109 (7)	0.0156 (8)	−0.0059 (7)	−0.0022 (7)	−0.0030 (6)
C14	0.0199 (9)	0.0153 (8)	0.0120 (8)	−0.0060 (7)	−0.0053 (7)	−0.0003 (6)
C15	0.0100 (8)	0.0148 (8)	0.0138 (8)	−0.0028 (6)	−0.0017 (6)	−0.0007 (6)
C16	0.0129 (9)	0.0186 (8)	0.0145 (8)	−0.0030 (7)	−0.0016 (7)	−0.0012 (7)
C17	0.0116 (9)	0.0227 (9)	0.0174 (9)	−0.0045 (7)	−0.0013 (7)	0.0052 (7)
C18	0.0141 (9)	0.0163 (8)	0.0270 (10)	−0.0057 (7)	−0.0035 (8)	0.0038 (7)
C19	0.0147 (9)	0.0151 (8)	0.0219 (9)	−0.0062 (7)	−0.0029 (7)	−0.0014 (7)
N1	0.0113 (7)	0.0118 (6)	0.0129 (7)	−0.0042 (5)	−0.0020 (5)	−0.0004 (5)
N2	0.0147 (8)	0.0116 (7)	0.0125 (7)	−0.0044 (6)	−0.0030 (6)	−0.0018 (5)
N3	0.0153 (8)	0.0146 (7)	0.0187 (7)	−0.0069 (6)	0.0005 (6)	−0.0023 (6)
N4	0.0155 (8)	0.0111 (6)	0.0124 (7)	−0.0049 (6)	−0.0030 (6)	−0.0001 (5)
N5	0.0146 (8)	0.0142 (7)	0.0160 (7)	−0.0065 (6)	−0.0029 (6)	0.0004 (5)
Cl1	0.0158 (2)	0.01449 (19)	0.0216 (2)	−0.00422 (16)	−0.00132 (17)	0.00282 (16)
Cl2	0.0160 (2)	0.0182 (2)	0.0178 (2)	−0.00561 (17)	−0.00165 (16)	−0.00182 (16)
O1	0.0217 (8)	0.0225 (7)	0.0175 (7)	−0.0062 (6)	−0.0017 (6)	−0.0028 (5)
O2	0.0234 (8)	0.0193 (7)	0.0194 (7)	−0.0083 (6)	−0.0002 (6)	−0.0040 (5)

### Geometric parameters (Å, º)

**Table:**

C1—N3	1.345 (2)	C12—C13	1.507 (2)
C1—C2	1.380 (3)	C13—N4	1.487 (2)
C1—H1	0.95	C13—H13A	0.99
C2—C3	1.387 (3)	C13—H13B	0.99
C2—H2	0.95	C14—N4	1.480 (2)
C3—C4	1.379 (3)	C14—C15	1.511 (2)
C3—H3	0.95	C14—H14A	0.99
C4—C5	1.393 (2)	C14—H14B	0.99
C4—H4	0.95	C15—N5	1.340 (2)
C5—N3	1.343 (2)	C15—C16	1.393 (2)
C5—C6	1.501 (2)	C16—C17	1.387 (3)
C6—N2	1.485 (2)	C16—H16	0.95
C6—H6A	0.99	C17—C18	1.393 (3)
C6—H6B	0.99	C17—H17	0.95
C7—N2	1.488 (2)	C18—C19	1.384 (2)
C7—C8	1.507 (2)	C18—H18	0.95
C7—H7A	0.99	C19—N5	1.342 (2)
C7—H7B	0.99	C19—H19	0.95
C8—N1	1.331 (2)	N2—H21	0.937 (18)
C8—C9	1.388 (2)	N2—H22	0.86 (2)
C9—C10	1.387 (2)	N4—H41	0.91 (2)
C9—H9	0.95	N4—H42	0.967 (19)
C10—C11	1.381 (2)	O1—H101	0.93 (3)
C10—H10	0.95	O1—H102	1.00 (4)
C11—C12	1.390 (2)	O2—H201	0.85 (3)
C11—H11	0.95	O2—H202	0.91 (3)
C12—N1	1.338 (2)		
			
N3—C1—C2	123.83 (18)	N4—C13—H13A	109.7
N3—C1—H1	118.1	C12—C13—H13A	109.7
C2—C1—H1	118.1	N4—C13—H13B	109.7
C1—C2—C3	118.32 (17)	C12—C13—H13B	109.7
C1—C2—H2	120.8	H13A—C13—H13B	108.2
C3—C2—H2	120.8	N4—C14—C15	111.73 (14)
C4—C3—C2	118.88 (17)	N4—C14—H14A	109.3
C4—C3—H3	120.6	C15—C14—H14A	109.3
C2—C3—H3	120.6	N4—C14—H14B	109.3
C3—C4—C5	119.23 (17)	C15—C14—H14B	109.3
C3—C4—H4	120.4	H14A—C14—H14B	107.9
C5—C4—H4	120.4	N5—C15—C16	122.97 (16)
N3—C5—C4	122.47 (16)	N5—C15—C14	117.68 (14)
N3—C5—C6	119.35 (15)	C16—C15—C14	119.28 (15)
C4—C5—C6	118.09 (16)	C17—C16—C15	118.87 (16)
N2—C6—C5	112.92 (14)	C17—C16—H16	120.6
N2—C6—H6A	109	C15—C16—H16	120.6
C5—C6—H6A	109	C16—C17—C18	118.78 (17)
N2—C6—H6B	109	C16—C17—H17	120.6
C5—C6—H6B	109	C18—C17—H17	120.6
H6A—C6—H6B	107.8	C19—C18—C17	118.13 (17)
N2—C7—C8	110.77 (13)	C19—C18—H18	120.9
N2—C7—H7A	109.5	C17—C18—H18	120.9
C8—C7—H7A	109.5	N5—C19—C18	123.98 (17)
N2—C7—H7B	109.5	N5—C19—H19	118
C8—C7—H7B	109.5	C18—C19—H19	118
H7A—C7—H7B	108.1	C8—N1—C12	118.13 (14)
N1—C8—C9	123.01 (15)	C6—N2—C7	111.76 (13)
N1—C8—C7	116.08 (14)	C6—N2—H21	106.6 (12)
C9—C8—C7	120.90 (15)	C7—N2—H21	105.3 (13)
C10—C9—C8	118.50 (16)	C6—N2—H22	107.3 (13)
C10—C9—H9	120.7	C7—N2—H22	108.9 (13)
C8—C9—H9	120.7	H21—N2—H22	117.1 (18)
C11—C10—C9	118.94 (16)	C5—N3—C1	117.25 (15)
C11—C10—H10	120.5	C14—N4—C13	112.47 (13)
C9—C10—H10	120.5	C14—N4—H41	108.2 (14)
C10—C11—C12	118.65 (16)	C13—N4—H41	107.4 (14)
C10—C11—H11	120.7	C14—N4—H42	112.1 (14)
C12—C11—H11	120.7	C13—N4—H42	106.6 (15)
N1—C12—C11	122.75 (16)	H41—N4—H42	110 (2)
N1—C12—C13	115.14 (14)	C15—N5—C19	117.26 (15)
C11—C12—C13	122.10 (15)	H101—O1—H102	102 (2)
N4—C13—C12	109.75 (13)	H201—O2—H202	115 (2)

### Hydrogen-bond geometry (Å, º)

**Table:**

*D*—H···*A*	*D*—H	H···*A*	*D*···*A*	*D*—H···*A*
O2—H201···Cl2	0.85 (3)	2.41 (3)	3.1844 (15)	152 (2)
O2—H202···N5	0.91 (3)	2.10 (3)	2.947 (2)	155 (2)
N2—H21···O2	0.94 (2)	1.89 (2)	2.804 (2)	165 (2)
N2—H22···Cl1^i^	0.86 (2)	2.31 (2)	3.1446 (17)	162.5 (17)
N4—H41···Cl2	0.91 (2)	2.25 (2)	3.1319 (17)	164.1 (19)
N4—H42···O1^i^	0.97 (2)	1.90 (2)	2.825 (2)	158 (2)
O1—H101···Cl1	0.93 (3)	2.43 (3)	3.2611 (15)	149 (2)
O1—H102···N3^ii^	1.00 (4)	1.93 (4)	2.920 (2)	172 (3)
C3—H3···Cl1^iii^	0.95	2.73	3.5702 (19)	148
C6—H6*A*···Cl1	0.99	2.8	3.751 (2)	161
C7—H7*A*···Cl1^iv^	0.99	2.78	3.7351 (18)	162
C10—H10···Cl1^v^	0.95	2.71	3.6469 (18)	168
C17—H17···O1^vi^	0.95	2.57	3.437 (2)	151

Symmetry codes: (i) *x*−1, *y*, *z*; (ii) *x*+1, *y*, *z*; (iii) *x*−1, *y*+1, *z*; (iv) −*x*+1, −*y*+1, −*z*+2; (v) *x*, *y*−1, *z*; (vi) −*x*+1, −*y*+2, −*z*+1.
